# CHOPCHOP v2: a web tool for the next generation of CRISPR genome engineering

**DOI:** 10.1093/nar/gkw398

**Published:** 2016-05-16

**Authors:** Kornel Labun, Tessa G. Montague, James A. Gagnon, Summer B. Thyme, Eivind Valen

**Affiliations:** 1 Computational Biology Unit, Department of Informatics, University of Bergen, 5008 Bergen, Norway; 2 Department of Molecular and Cellular Biology, Harvard University, Cambridge, MA 02138, USA; 3 Sars International Centre for Marine Molecular Biology, University of Bergen, 5008 Bergen, Norway

## Abstract

In just 3 years CRISPR genome editing has transformed biology, and its popularity and potency continue to grow. New CRISPR effectors and rules for locating optimum targets continue to be reported, highlighting the need for computational CRISPR targeting tools to compile these rules and facilitate target selection and design. CHOPCHOP is one of the most widely used web tools for CRISPR- and TALEN-based genome editing. Its overarching principle is to provide an intuitive and powerful tool that can serve both novice and experienced users. In this major update we introduce tools for the next generation of CRISPR advances, including Cpf1 and Cas9 nickases. We support a number of new features that improve the targeting power, usability and efficiency of CHOPCHOP. To increase targeting range and specificity we provide support for custom length sgRNAs, and we evaluate the sequence composition of the whole sgRNA and its surrounding region using models compiled from multiple large-scale studies. These and other new features, coupled with an updated interface for increased usability and support for a continually growing list of organisms, maintain CHOPCHOP as one of the leading tools for CRISPR genome editing. CHOPCHOP v2 can be found at http://chopchop.cbu.uib.no

## INTRODUCTION

The discovery and adoption of the CRISPR bacterial system for genome editing has led to a revolution in biology: targeted mutations are now possible in a multitude of organisms, including many not previously amenable to genetic manipulation. This has both transformed our approach to answering biological questions and unlocked the possibility of correcting human genetic diseases.

**Figure 1.**
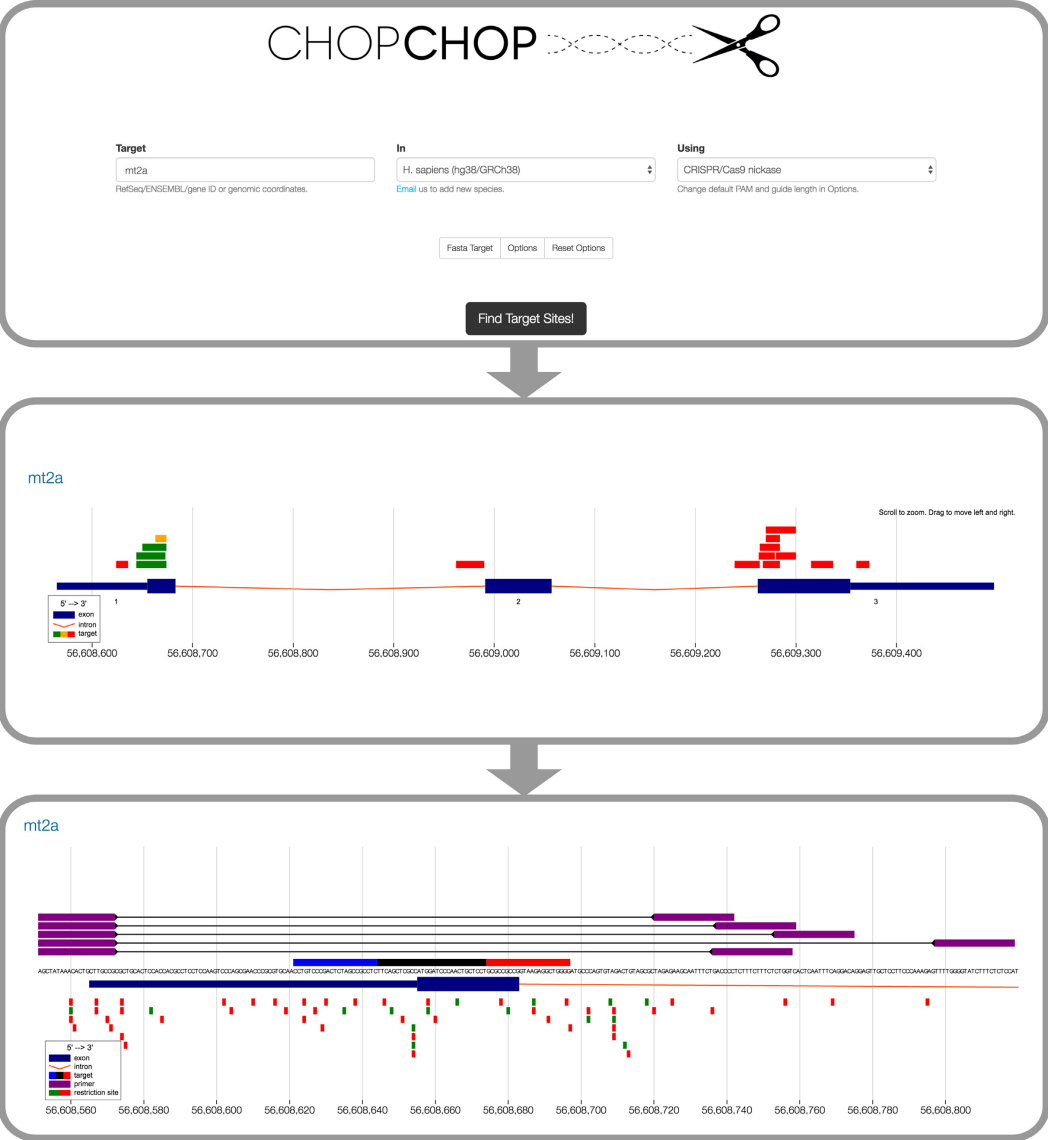
The workflow of CHOPCHOP in Cas9 nickase mode. The CHOPCHOP homepage (upper box) allows three types of input (DNA sequence, genomic coordinates or gene IDs) with default parameters optimized for novice users. For experienced users, a number of options for Cas9, Cas9 nickase, Cpf1 and TALEN mode can be revealed. The results of the search (middle box) are displayed across the gene, genomic region or DNA sequence, depending on the input format. The target color indicates the quality of each sgRNA or nickase pair (green [best] to red [worst]). The graphic representation of the search area is complemented by an interactive table below (not shown). Each sgRNA or nickase pair can be explored in greater detail (lower box) with annotated primer candidates and restriction sites, and information about any off-targets (not shown). Nickases are displayed in red and blue with the intermediate region in black.

Originally harnessed from the *Streptococcus pyogenes* type II system (1–3), CRISPR genome editing is based on a two-component system: a Cas9 nuclease and a single guide RNA (sgRNA), which directs the nuclease to a specific site in the genome. In the presence of the sgRNA, Cas9 locates the target site and makes a double-strand break (DSB). The DSB is repaired by the host non-homologous end-joining pathway, but often the repair is imperfect, creating indels and in many cases frameshift mutations. Since the technology's inception, research to improve the technology has focused on two main challenges: optimization of cutting efficiency and specificity of cutting. A substantial portion of sgRNAs designed for a given gene will produce a low or zero cutting rate, and many sgRNAs have the capacity to bind promiscuously in the genome, which can lead to off-target mutagenesis (4–10). To address these issues, research has focused on identifying the sequence features that contribute to effective (and ineffective) sgRNAs (11–16), as well as the development of new CRISPR variants that expand the targeting range and specificity of the nuclease (17–20). With the contribution of so many factors to optimum sgRNA target selection, it has become necessary to use software to aid selection of CRISPR target sites for experiments. CHOPCHOP (21) provides an intuitive online environment for target selection that optimizes efficiency and specificity according to the latest large-scale studies, as well as performing primer design and restriction site identification, all in a user-friendly, graphical interface (Figure 1). This new update of CHOPCHOP provides additional flexibility by offering new options for sgRNA design, as well as additional metrics by which sgRNA targets are scored and ranked.

## IMPROVEMENTS IN THE 2016 RELEASE

**Figure 2.**
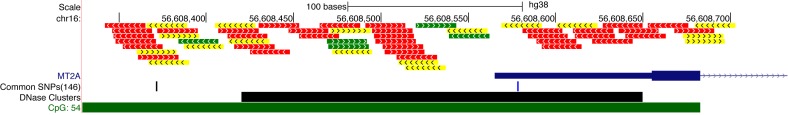
CHOPCHOP results can be exported to the UCSC browser with a single click. Here, the sgRNAs (in this example in promoter-targeting mode) are viewed in the context of the genome. The tracks displayed in this example are DNase sensitive regions, common SNPs and CpG islands.

CHOPCHOP accepts multiple input formats (gene identifiers, genomic coordinates and pasted sequences) for a wide range of organisms, and provides instant, visual output as well as downloadable data (GenBank, text tables and FASTA files). In this new version users can also view the output data in the UCSC browser (22) with a single click, enabling results to be viewed in the context of annotated genomic features, such as transcription factor binding sites and chromatin architecture and accessibility (Figure 2).

CHOPCHOP offers flexible targeting to sub-regions of protein-coding and non-coding genes, including coding regions, UTRs, splice sites and individual exons. In this new version we also offer a promoter-targeting mode (Figure 2) for experiments such as down- or upregulating gene expression using catalytically dead Cas9 (dCas9) or transcriptionally active dCas9 (e.g. dCas9-VP64), respectively (23–25). CHOPCHOP determines potential off-target sites for all sgRNAs using Bowtie (26) and automatically generates primers for target sites using Primer3 (27). The length and annealing temperature of the primers, as well as the size of the amplicon, can be specified. CHOPCHOP visualizes all elements in a dynamic visual interface that includes information about restriction sites, which can be used for downstream validation.

In addition to these improvements, the new iteration of CHOPCHOP introduces the following major new features.

### Support for a new generation of CRISPR effectors

The most widely used CRISPR effector is Cas9, derived from the type II *S. pyogenes* system. While the RNA-mediated targeting of Cas9 offers great versatility in selecting a target site, a limiting factor is the requirement for an NGG protospacer adjacent motif (PAM) motif adjacent to the target. The occurrence of this motif is not rare in most genomes, but it imposes a restriction that can be inimical to achieving the high genomic precision required for certain experiments, or for targeting small genes. The new generation of CRISPR effectors vastly expands the universe of viable targets by offering alternative PAM motifs (Supplementary Table S1, Supplementary Figures S1 and 2). CHOPCHOP now provides support for alternative CRISPR effectors, including Cpf1 from *Acidaminococcus*, which utilizes an AT-rich PAM (17) and Cas9 homologs from *S. pyogenes, Streptococcus thermophilus, Staphylococcus aureus* and *Neisseria meningitidis* (28). In addition, CHOPCHOP also accepts user-defined custom PAMs that can be anchored to the 5′ (Cpf1) or 3′ (Cas9) end of the sgRNA. This field accepts the standard IUPAC nucleotide alphabet (29), including ambiguity codes. CHOPCHOP therefore provides support for the sequence requirements of any currently known CRISPR effector and enables immediate adoption of any new CRISPR effectors. This greatly increases the targeting range of CRISPR experiments that can be designed with CHOPCHOP, including improved targeting of AT-rich genomes such as *Plasmodium falciparum* (Supplementary Figure S2).

### New rules for optimizing cutting efficiency

CRISPR sgRNAs can be ranked by 2 criteria: (i) efficiency—the likelihood that the particular sgRNA facilitates cutting, and (ii) specificity—the likelihood that the sgRNA binds off-target sites.

The initial release of CHOPCHOP provided two simple metrics for efficiency based on experimental studies. First, the GC-content of the sgRNA—ideally between 40 and 80%—and second, whether the sgRNA contains a G at position 20 (11,30). Since the initial release of CHOPCHOP, several refinements have been proposed. A study from Doench *et al*. produced a large dataset to calculate efficiencies across a wide range of sgRNAs (14), and the rules for computationally-aided sgRNA design were recently further refined by the same group (13). Moreno-Mateos *et al*. conducted similar screens and found that sgRNA stability, which depends on guanine enrichment and adenine depletion, was a major determinant of sgRNA efficiency (12). Chari *et al*. conducted a study exploiting the bias of lentiviral integration into transcriptionally active regions, which: (i) revealed that accessible DNA is more amenable to cutting with Cas9; (ii) separated the influence of DNA accessibility and sequence composition on sgRNA efficiency. CHOPCHOP users can now view results in the UCSC browser (22) in the context of DNase I hypersensitivity sites to predict accessible DNA regions (Figure 2). Finally, a meta study by Xu *et al*. compiled the sequence specificities across multiple datasets to build an aggregate model (15). We have implemented all of these metrics in the new release to give the user a broad selection of metrics to choose from (the default is the Xu *et al*. metric). Using these methods, CHOPCHOP can now score every sgRNA using position-specific scoring matrices or support vector machines that consider each individual position of the sgRNA as well as the sequence downstream of the PAM and upstream of the binding site. In the results table this score is reported as the ‘efficiency score’.

Other factors also play a role in whether an sgRNA is likely to cut at its intended target. Recently, we and others showed that self-complementarity of the sgRNA can inhibit its efficient incorporation into the effector complex (12,31). CHOPCHOP now includes the basic self-complementarity score of the Thyme *et al*. study (31), which computes the number of potential 4 bp stems within the sgRNA and between the sgRNA and the backbone. The user can therefore opt to avoid sgRNAs with self-complementarity using this option.

### Strategies to increase specificity

A significant challenge in CRISPR experiments is the possibility of inducing cleavage at sites other than the intended target. An emerging tool to alleviate this problem is the paired nickase approach (32). Unlike natural CRISPR effectors, nickases have been modified to cut only one DNA strand. In order to create a DSB, a pair of nickases must be targeted to opposite strands and bind within 10–31 bp of each other (32). These requirements vastly reduce the likelihood of creating off-target DSBs, and CHOPCHOP has now added support for paired nickase experiments. In this mode, sites on opposite strands within a specified distance (either default or user-defined) are paired as potential nickase sites. For these sites, in addition to the default off-target search, each pair of sites is evaluated for off-targets where binding and cutting would result in a DSB. Nickase sites are visualized with two CRISPR targets surrounding a ‘break’ region (Figure 1).

Recent studies have highlighted the need to search for more than two mismatches when identifying off-targets (10) so CHOPCHOP now counts off-targets with up to three mismatches. While off-targets with more than three mismatches have been reported (10), evidence suggests that almost all predicted sites of four mismatches or more are not cleaved (10) and therefore the vast majority of such predicted sites would be misleading and unnecessarily time-consuming to search for during sgRNA selection.

Another strategy that has been shown to decrease off-target cleavage is the use of truncated sgRNAs (10,20). Besides increasing specificity, 5′ shortening of the customary 20 bp also increases the targeting range. The new version of CHOPCHOP therefore provides support for sgRNAs of user-defined lengths.

Thus, this version of CHOPCHOP supports a number of new features that: (i) improve the ability to target a broader range of sequences, and (ii) more thoroughly predict potential off-target sites in the genome. For an example of the increased targeting range and additions to the scoring system between the old and new versions of CHOPCHOP, see Supplementary Figure S3 and Table S2.

### New genomes

In addition to a new range of features, CHOPCHOP strives to accommodate all requests for new genomes and gene annotation sets. So far we have incorporated all inquiries received, and CHOPCHOP now supports a total of 32 organisms. Furthermore, all genomes have been updated to their most recent assemblies and suggestions for new species can easily be submitted through a link on the main page.

## DISCUSSION AND FUTURE DEVELOPMENTS

The overarching principle of CHOPCHOP is to provide an intuitive and powerful tool that can serve first time as well as experienced users. The basic mode offers optimized defaults for the basic user, while more advanced users can select from a wide range of options curated from the literature by their relevance and utility. All options are presented in a tabulated and organized manner to help users quickly visualize and evaluate options when designing CRISPR experiments.

This release retains the general layout of the previous release, but updates the visual profile to a modern look and to accommodate new features. The site is now mobile and tablet friendly, and to streamline the user's experience we use cookies to remember the selection of species and targeting options for subsequent searches. All reported bugs have been fixed, and the implementation is now optimized for future development to facilitate both rapid adoption of any future effectors and new targeting data from large-scale studies. This major update maintains CHOPCHOP as one of the most easy-to-use, versatile and powerful CRISPR targeting tools available.

## Supplementary Material

SUPPLEMENTARY DATA
