# Supplementary material for: CHOPCHOP v2: a web tool for the next generation of CRISPR genome engineering
Source: Nucleic Acids Res. 2016 May 16;44(Web Server issue):W272–6. doi: 10.1093/nar/gkw398 (PMC4987937; doi:10.1093/nar/gkw398)
Supplement: SUPPLEMENTARY DATA [file supp_44_W1_W272__index.html]

CHOPCHOP v2: a web tool for the next generation of CRISPR genome engineering — SUPPLEMENTARY DATA 

# CHOPCHOP v2: a web tool for the next generation of CRISPR genome engineering

## SUPPLEMENTARY DATA

- SUPPLEMENTARY DATA
