# Supplementary material for: CHOPCHOP v2: a web tool for the next generation of CRISPR genome engineering
Source: Nucleic Acids Res. 2016 May 16;44(Web Server issue):W272–6. doi: 10.1093/nar/gkw398 (PMC4987937; doi:10.1093/nar/gkw398)
Supplement: SUPPLEMENTARY DATA [file supp_gkw398_nar-00411-web-b-2016-File004.pdf]

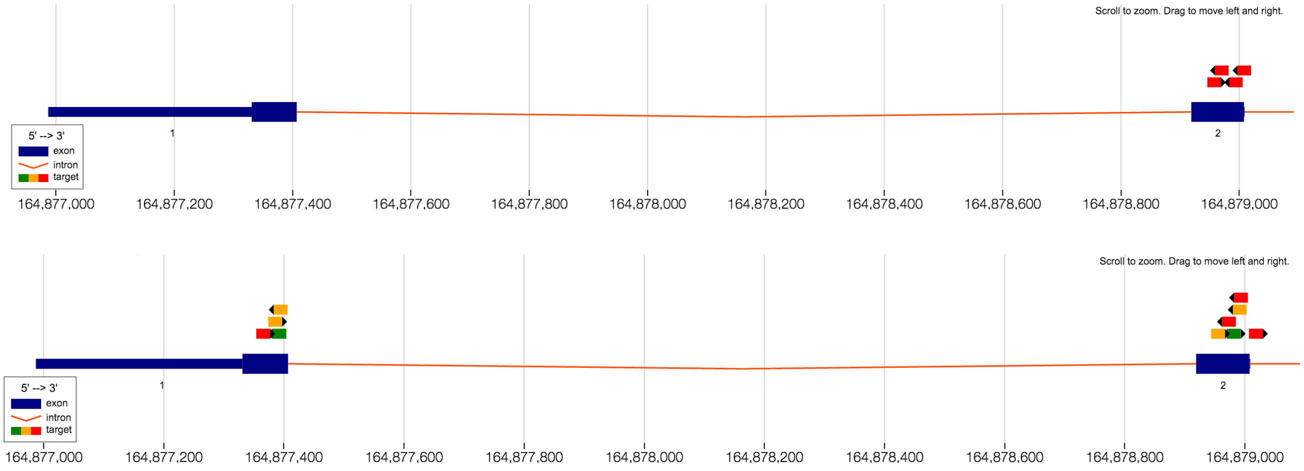

Supplementary Figure 1: Increased range of targeting in CHOPCHOP v2. Targeting the zebrafish gene *Apela* with the SpCas9 CRISPR effector (NGG PAM, Xu et al. scoring) yields no good sgRNAs (upper panel, only red). Targeting with a modified Cas9 (NGA PAM, lower panel) yields two good sgRNAs (green), and 4 adequate sgRNAs (yellow).

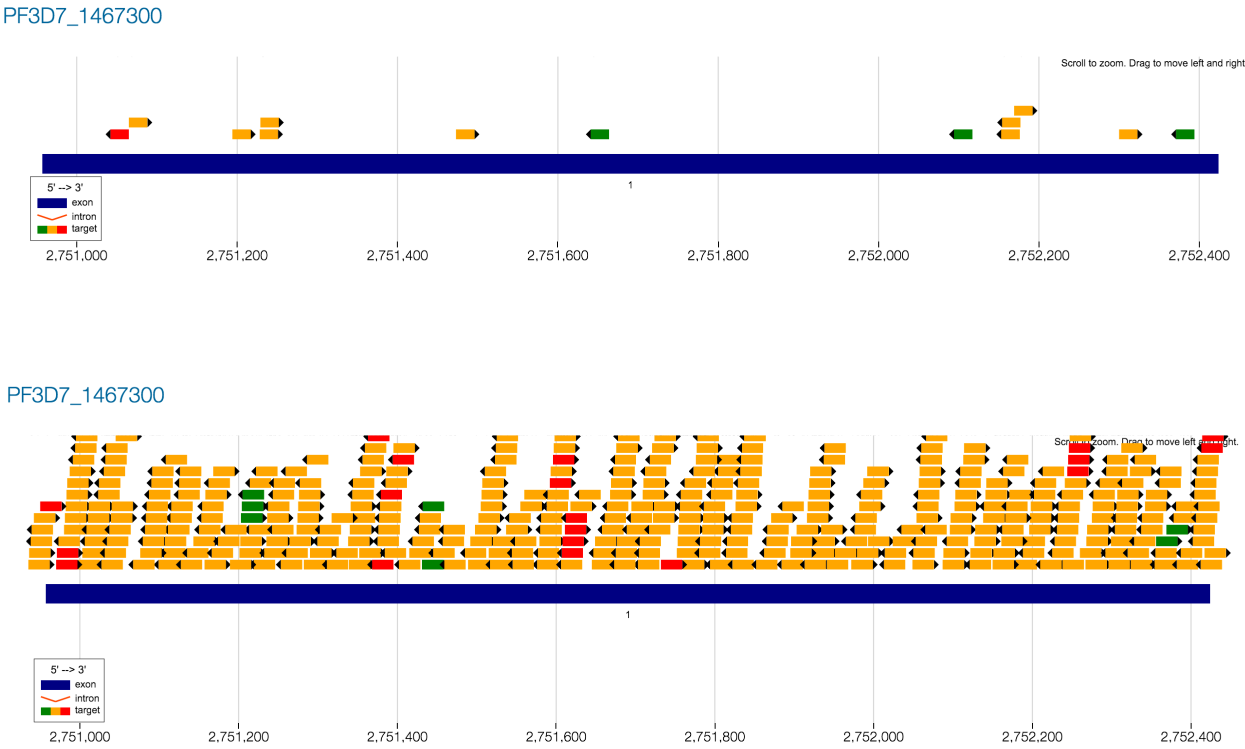

Supplementary Figure 2: Targeting genes in an AT-rich genome with Cpf1. The GC-content of *P. falciparum* is only 23%. The standard Cas9 (upper panel) requires a G-rich PAM site (NGG) which may severely impede the search for sgRNAs in such genomes. In these cases alternative CRISPR effectors like Cpf1 that have a T-rich PAM (TTN) enables efficient targeting (lower panel).

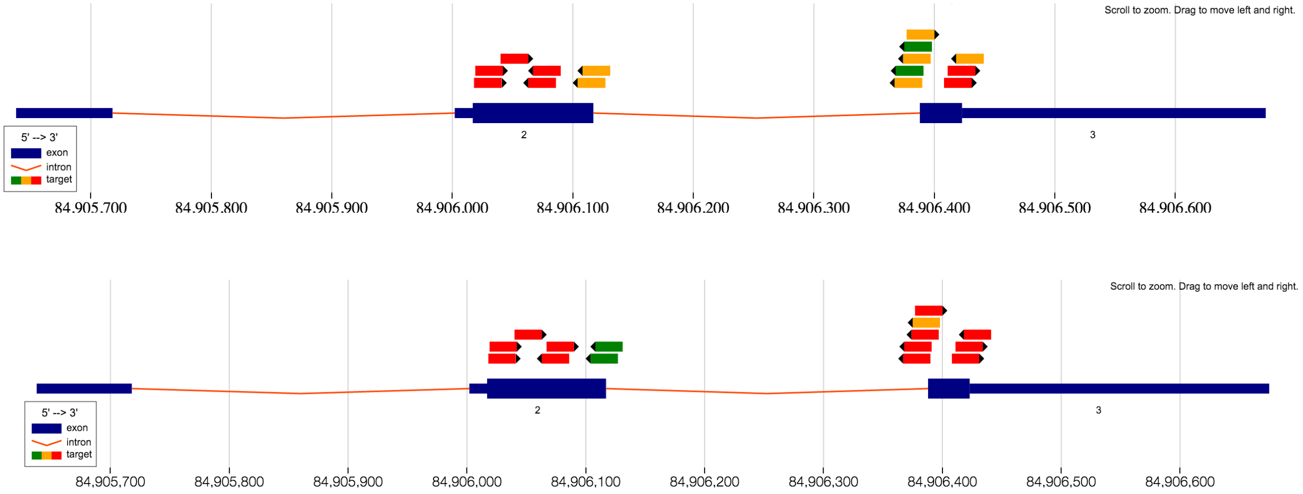

Supplementary Figure 3: **Comparison of scoring in CHOPCHOP v1 versus v2.** Example human gene *TMSB10* targeted by CHOPCHOP v1 (upper panel) and v2 (lower panel, Xu scoring scheme). Due to a large number of 3 mismatch off-targets for the top 2 sgRNAs in v1, those sgRNAs are now demoted in v2. Conversely, in v2, two sgRNAs lacking a G at position 20 are promoted due to having reasonable efficiency under the Xu model and no predicted off-targets. Scoring for individual sgRNAs and a comparison of the sgRNAs is found in Supplementary Table 2.

CRISPR effector	PAM	Publication
Streptococcus pyogenes	NGG-3'	Jinek, M., Chylinski, K., Fonfara, I., Hauer, M., Doudna, J.A., Charpentier, E. (2012) A programmable dual-RNA-guided DNA endonuclease in adaptive bacterial immunity. Science 337(6096): 816–821.
Streptococcus pyogenes	NAG-3'	Kleinstiver,B.P., Pattanayak,V., Prew,M.S., Tsai,S.Q., Nguyen,N.T., Zheng,Z. and Keith Joung,J. (2016) High-fidelity CRISPR-Cas9 nucleases with no detectable genome-wide off-target effects. Nature 529, 490–495
Streptococcus pyogenes	NGA-3'	Kleinstiver,B.P., Pattanayak,V., Prew,M.S., Tsai,S.Q., Nguyen,N.T., Zheng,Z. and Keith Joung,J. (2016) High-fidelity CRISPR-Cas9 nucleases with no detectable genome-wide off-target effects. Nature 529, 490–495
Streptococcus thermophilus	NNAGAAW-3'	Sapranauskas, R., Gasiunas, G., Fremaux, C., Barrangou, R., Horvath, P., & Siksnys, V. (2011). The Streptococcus thermophilus CRISPR/Cas system provides immunity in Escherichia coli. Nucleic Acids Research, 39(21), 9275–9282
Neisseria meningitidis	NNNNGMTT-3'	Hou, Z., Zhang, Y., Propson, N. E., Howden, S. E., Chu, L.-F., Sontheimer, E. J., & Thomson, J. A. (2013). Efficient genome engineering in human pluripotent stem cells using Cas9 from Neisseria meningitidis. Proceedings of the National Academy of Sciences of the United States of America, 110(39), 15644–15649
Staphylococcus aureus	NNGRRT-3'	Ran, F. A., Cong, L., Yan, W. X., Scott, D. A., Gootenberg, J. S., Kriz, A. J., … Zhang, F. (2015). In vivo genome editing using Staphylococcus aureus Cas9. Nature, 520(7546), 186–191
Acidaminococcus, Lachnospiraceae	5'-TTTN	Zetsche,B., Gootenberg,J.S., Abudayyeh,O.O., Slaymaker,I.M., Makarova,K.S., Essletzbichler,P., Volz,S.E., Joung,J., van der Oost,J., Regev,A., et al. (2015) Cpf1 Is a Single RNA-Guided Endonuclease of a Class 2 CRISPR-Cas System. Cell 163.3 (2015): 759-771.
Prevotella, Francisella	5'-TTN	Zetsche,B., Gootenberg,J.S., Abudayyeh,O.O., Slaymaker,I.M., Makarova,K.S., Essletzbichler,P., Volz,S.E., Joung,J., van der Oost,J., Regev,A., et al. (2015) Cpf1 Is a Single RNA-Guided Endonuclease of a Class 2 CRISPR-Cas System. Cell 163.3 (2015): 759-771.

Supplementary Table 1: Overview of the CRISPR effectors and the PAMs supported by CHOPCHOP. In addition, CHOPCHOP supports custom PAMs at either the 5' or 3' end which can be specified with the IUPAC alphabet.

Ranking	Target sequence	Genomic location	Exon	Strand	GC (%)	Off-targets		
						0	1	2
1	TGGCTGTGGAGAGCGGGAAGCGG	chr2:84906369	3	"-"	70	0	0	0
2	TGCTCAATGGCTGTGGAGAGCGG	chr2:84906376	3	"-"	61	0	0	0
3	GCACACTCACTCTCTTTGGTCGG	chr2:84906105	2	"-"	57	0	0	0
4	CGAGGCACACTCACTCTCTTTGG	chr2:84906109	2	"-"	57	0	0	0
5	GCTCTCCACAGCCATTGAGCAGG	chr2:84906378	3	"+"	61	0	0	0
6	GGCTGTGGAGAGCGGGAAGCGGG	chr2:84906368	3	"-"	74	0	0	2
7	GCTCAATGGCTGTGGAGAGCGGG	chr2:84906375	3	"-"	65	0	0	2
8	AGGAAATCCTCCAGGATCTTAGG	chr2:84906419	3	"-"	48	0	0	2
9	GAAATTTCCTAAGATCCTGGAGG	chr2:84906412	3	"+"	43	0	1	1
10	GGAAATCGCCAGCTTCGATAAGG	chr2:84906041	2	"+"	52	0	1	0
11	TGGCAGACAAACCAGACATGGGG	chr2:84906019	2	"+"	57	0	3	0
12	GGCAGACAAACCAGACATGGGGG	chr2:84906020	2	"+"	61	0	3	0
13	AGTGAAATTTCCTAAGATCCTGG	chr2:84906409	3	"+"	39	0	1	2
14	CGTCTCCGTTTTCTTCAGCTTGG	chr2:84906064	2	"-"	52	1	0	1
15	GCTGAAGAAAACGGAGACGCAGG	chr2:84906068	2	"-"	57	1	0	1

Ranking	Target sequence	Genomic location	Exon	Strand	GC (%)	Self-complementarity	Off-targets				Efficiency
							0	1	2	3	
1	GCACACTCACTCTCTTTGGTCGG	chr2:84906105	2	"-"	57	1	0	0	0	0	0.41
2	CGAGGCACACTCACTCTCTTTGG	chr2:84906109	2	"-"	57	0	0	0	0	0	0.31
3	TGCTCAATGGCTGTGGAGAGCGG	chr2:84906376	3	"-"	61	0	0	0	0	2	0.58
4	GCTCTCCACAGCCATTGAGCAGG	chr2:84906378	3	"+"	61	1	0	0	0	5	0.59
5	GGAAATCGCCAGCTTCGATAAGG	chr2:84906041	2	"+"	52	1	0	1	0	3	0.53
6	CGTCTCCGTTTTCTTCAGCTTGG	chr2:84906064	2	"-"	52	0	1	0	1	2	0.4
7	TGGCAGACAAACCAGACATGGGG	chr2:84906019	2	"+"	57	0	0	3	0	2	0.62
8	AGGAAATCCTCCAGGATCTTAGG	chr2:84906419	3	"-"	48	2	0	0	2	5	0.29
9	GCTGAAGAAAACGGAGACGCAGG	chr2:84906068	2	"+"	57	2	1	0	1	6	0.67
10	GGCAGACAAACCAGACATGGGGG	chr2:84906020	2	"+"	61	0	0	3	0	4	0.61
11	AGTGAAATTTCCTAAGATCCTGG	chr2:84906409	3	"+"	39	0	0	0	2	6	0.47
12	GAAATTTCCTAAGATCCTGGAGG	chr2:84906412	3	"+"	43	1	0	1	1	8	0.59
13	GCTCAATGGCTGTGGAGAGCGGG	chr2:84906375	3	"-"	65	0	0	0	2	9	0.48
14	TGGCTGTGGAGAGCGGGAAGCGG	chr2:84906369	3	"-"	70	0	0	0	0	13	0.67
15	GGCTGTGGAGAGCGGGAAGCGGG	chr2:84906368	3	"-"	74	0	0	0	2	10	0.54

Ranking in version 1	Ranking in version 2	Cause of difference
1	14	Although it has a high efficiency score, CHOPCHOP v2 found 13 off-targets with 3 mismatches for this sgRNA
2	3	This sgRNA has a reasonable efficiency score, but CHOPCHOP v2 found 2 off-targets with 3 mismatches
3	1	These sgRNAs have a modest efficiency score, but they have no predicted off-targets so are considered the best. Please note: sgRNA #1 (in v2) has one 4 nt region of self-complementarity, but since this is a novel scoring metric, it currently carries only a small penalty.
4	2	
5	4	CHOPCHOP v2 is more restrictive about off-targets and considers 5 off-targets with 3 mismatches to be a poor sgRNA
6	15	CHOPCHOP v2 demoted these sgRNAs due to the large number of 3 mismatch off-targets
7	13	
8	8	CHOPCHOP v2 identified additional 3 mismatch off-targets for this sgRNA, as well as incidents of self-complementarity
9	12	While some of these sgRNAs have reasonable efficiency scores, all of them have too many off-targets to be considered adequate
10	5	
11	7	
12	10	
13	11	
14	6	
15	9	

Supplementary Table 2: Results from CHOPCHOP v1 (upper table) and v2 using the Xu et al. model for scoring (middle table) on the human gene TMSB10 displayed in Supplemental Figure 3. A comparison and explanation for the differences in ranking between the two versions can be found in the lower table.
